# The Emerging Role of m6A Modification in Endocrine Cancer

**DOI:** 10.3390/cancers15041033

**Published:** 2023-02-06

**Authors:** Xiaoyu Ji, Zhiyuan Wang, Wei Sun, Hao Zhang

**Affiliations:** Department of Thyroid Surgery, The First Hospital of China Medical University, No. 155 Nanjing North Street, Shenyang 110001, China

**Keywords:** endocrine cancer, m6A, methyltransferase, demethylase

## Abstract

**Simple Summary:**

Endocrine tumors often cause hormonal imbalances and sometimes hormone syndrome. Generally, the prognosis of patients with metastatic endocrine malignancies is poor due to recurrence and treatment failure. Aberrant expression of m6A may disrupt the development and occurrence of malignancies. We provide a theoretical foundation for the development of new diagnostic markers and therapeutic techniques based on m6A alteration and regulators in endocrine cancers.

**Abstract:**

With the development of RNA modification research, N6-methyladenosine (m6A) is regarded as one of the most important internal epigenetic modifications of eukaryotic mRNA. It is also regulated by methylase, demethylase, and protein preferentially recognizing the m6A modification. This dynamic and reversible post-transcriptional RNA alteration has steadily become the focus of cancer research. It can increase tumor stem cell self-renewal and cell proliferation. The m6A-modified genes may be the primary focus for cancer breakthroughs. Although some endocrine cancers are rare, they may have a high mortality rate. As a result, it is critical to recognize the significance of endocrine cancers and identify new therapeutic targets that will aid in improving disease treatment and prognosis. We summarized the latest experimental progress in the m6A modification in endocrine cancers and proposed the m6A alteration as a potential diagnostic marker for endocrine malignancies.

## 1. Introduction

Endocrine glands include the thyroid gland, adrenal gland, pancreas, parathyroid gland, ovary, and pituitary gland. Malignant tumors occurring in endocrine glands are called endocrine tumors [1,2]. Endocrine tumors often cause hormonal imbalances and sometimes hormone syndrome [3,4]. Although some endocrine tumors are rare in clinical practice, they can be fatal. Generally, the prognosis of patients with metastatic endocrine malignancies is poor due to recurrence and treatment failure [5].

In the 1970s, m6A was identified in eukaryotic messenger RNA (mRNA) [6,7] and viral nuclear RNA [8,9] for the first time. In eukaryotic mRNA, it is the most common dynamic internal genetic change, influencing RNA maturation, transcription, localization, translation, and metabolism [10]. It regulates many physiological and pathological processes [7]. The m6A modification has been proven to contain a preserved sequence “RRACH” ([G A] m6AC [U A C]) through m6A antibody-based affinity enrichment tests, high-throughput m6A sequencing, and methylated RNA m6A immunoprecipitation sequencing (MeRIP/m6A-seq) [11,12,13]. The m6A modification is not a random process. It is translated close to the 5’UTR or in the long exon, and it is enriched close to the termination codon and 3’UTRs [11,12]. Several enzymes catalyze the m6A modification, such as the m6A methyltransferase complex (*MTC*), also known as the m6A“writer”. The enzyme that removes m6A is called demethylase, also called the m6A“eraser” [8,9]. The enzyme that recognizes m6A and regulates the functions of activated m6A is called the m6A“reader” [10]. The expression of genes for writers, erasers, and readers influences m6A levels [11]. Aberrant m6A modification is associated with an increased risk of cancer.

Multiple studies have established a link between m6A variables and the development of endocrine cancers. Recent discoveries in relation to the m6A modification in endocrine cancers are discussed in this review.

## 2. M6A Methyltransferase

### 2.1. Writers

**Figure 1:**
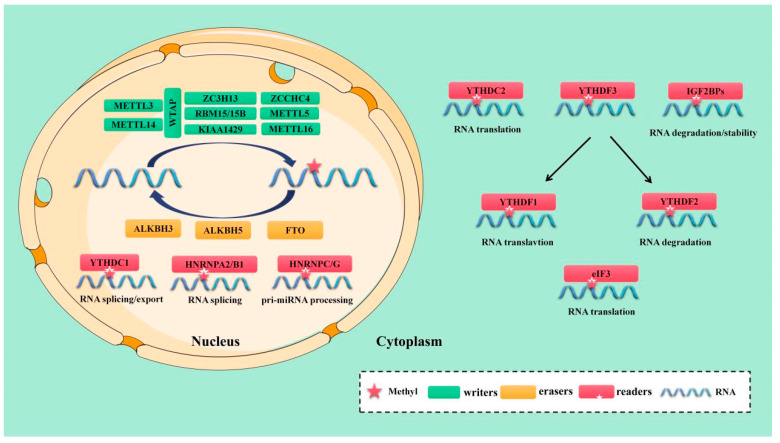
The molecular mechanism of m6A mutation. m6A is installed by “writers” (METTL3/5/14/16, WTAP, RBM15/15B, KIAA1429, ZC3H13 and ZCCHC4,), removed by “erasers”(FTO, ALKBH5, and ALKBH3, termed “erasers”), and recognized by “readers”(YTHDC1/2, YTHDF1/2/3, IGF2BP1/2/3, HNRNPA2/B1, HNRNPC/G, and eIF3, termed “readers”.

The m6A methyltransferase of RNA consists of “write in” proteins, including methyltransferase-like 3/5/14/16 (*METTL3/5/14/16*), the RNA binding motif protein 15/15B (*RBM15/15B*), Wilm’s tuner 1-associated protein (*WTAP*), Vir-like m6A methyltransferase-associated protein (*VIRMA*, also called *KIAA1429*), Zinc finger CCCH-type containing thirteen (*ZC3H13*) and Zinc finger CCHC-type containing four (*ZCCHC4*). The protein-based *MTC* is responsible for m6A installation [12] (Figure 1).

Methyltransferase-like 3 (*METTL3*) is an S-adenosyl methyl (*SAM*) binding protein. In 1997, it was found that *METTL3* is the main methyltransferase of m6A methylation and the most important component of the m6A *MTC*. Its aberrant expression can potentially alter the total methylation level of m6A [13]. *METTL14* is another dynamic component of the m6A *MTC*. As the structural scaffold of *METTL3*, *METTL14* can recognize specific RNA sequences (“RRACH”) and stabilize the *MTC* structure [13,14,15]. Both *METTL3* and *METTL14* are found in the nucleus as part of the central *MTC* and function to stabilize the complex [13]. They both cause m6A alteration in a one-to-one proportion. [13]. However, the only *METTL3* serves as catalyst. To create S-adenosyl homocysteine, its internal *SAM* binding domain catalyzes the transfer of methyl from *SAM* to an adenine base in RNA (*SAH*). *METTL16* was proposed in 2017 as an independent mRNA methyltransferase. Given that its binding site does not overlap with that of the *METTL3*/*METTL14* methylation complex, *METTL16* may function to control the stability and splicing of mRNA [16,17]. Overexpression of the *METTL16* construct with a mutant catalytic domain initiated the splicing process [18]. In addition, it facilitated the attachment of m6A to the 3’UTR of mRNA and A43 to U6 MicroRNA (miRNA). A43 has been found in the ubiquitous U6”ACAGAGA”box and is known to regulate tumorigenesis by targeting forward mRNAs and non-coding RNA [19]. A previous study reported that *METTL3/16* is a multifunctional enzyme with non-catalytic activity. In the absence of enzyme cofactors, the m6A writer acts as a reader and combines with the unmodified substrate constitutively to trigger the non-catalytic function [19]. A novel methyltransferase called *METTL5* was recently discovered and is responsible for altering the 18S Ribosomal RNA (rRNA) m6A [20,21]. Like the *METTL3*/*METTL14* complex, *METTL5* is a co-activator of the TRNA methyltransferase activator subunit 11-2 (*TRMT112*). *METTL5* can form heterodimers with *TRMT112*, which improves metabolic durability and the modified region of 18S rRNA precursors and mature forms [20].

The adaptor protein *WTAP* interacts with *METTL3* and *METTL14*. Although it has no catalytic activity, it stabilizes the central complex. By attracting *METTL3* and *METTL14* to nuclear speckles, it mainly enhances the implantation of m6A [22,23]. *METTL3* interacts with RNA-binding motif protein 15 (*RBM15*) and *RBM15B* in a *WTAP*-dependent way, directing these two proteins to particular RNA locations for methylation, but they do not have any enzymatic activity themselves [24,25]. In addition, immunoprecipitation studies have identified 26 core interaction factors between a large number of direct repeat erythroid-definitive (*DREDs*) of *WTAP*-binding proteins, and more than 100 proteins may bind to *METTL3* or *METTL14* [26]. *MTC* remains in the nuclear speckles after *ZC3H13* interacts with *WTAP* via its low-complexity domain, which enhances its catalytic function [25,27]. After enlisting *MTC*, *VIRMA* discovered mRNA methylation close to the 3’ UTR and termination codon areas. Cleavage and polyadenylation-specific factor bundles 5 (*CPSF5*) and 6 (*CPSF6*) can also interact with it [28]. Recently, *ZCCHC4* was discovered to function as a new methyltransferase that regulates the distribution and overall translation of rRNA ribosomal subunits by altering 28S rRNA [29].

### 2.2. Erasers

The enzyme known as m6A demethylase, which controls the reversible and dynamic modification of m6A, is referred to as an “eraser” [30,31]. The amount of m6A in cells is modulated by the m6A demethylase and m6A tags from mRNA can be removed selectively through complex intermediary processes, thus affecting several biological processes of tumor cells. m6A demethylase is mainly composed of fat mass and obesity-associated protein (*FTO*) [31] and Alk B homolog 5 (*ALKBH5*) [30]. These two proteins are predominantly found in the nucleus and belong to the α- ketoglutarate-dependent dioxygenase family, with Fe (II) and α-ketoglutarate catalyzing the demethylation of m6A in a dependent manner. Studies have shown that *FTO* was the first protein that can catalyze m6A demethylation [31]. Variations in the *FTO* gene have been linked to weight gain, short stature, and other health issues [32,33,34]. *ALKBH5*, the second RNA demethylase identified, can oxidize and reverse the m6A modification. *ALKBH5* directly removes the methyl group from m6A-methylated adenosine, in contrast to the oxidative demethylation of *FTO* [35,36]. Moreover, recent research suggests that *ALKBH3* is a novel m6A-modified demethylase. *ALKBH3*’s new substrate is m6A in mammalian transfer RNA (tRNA). *ALKBH3* changes tRNA more frequently than mRNA and rRNA [37,38] (Figure 1).

### 2.3. Readers

The dynamic and reversible regulation of the m6A modification is driven by the functional interplay between the m6A writer and the eraser. However, different readers must recognize m6A for different downstream biological functions. The m6A reader can bind to RNA that contains m6A thanks to a conservative m6A binding domain, referred to as YTH domain family protein 1 (*YTHDF1/2/3*), YTH domain containing 1 (*YTHDC1/2*), the heterologous nuclear ribonucleoprotein (*HNRNP*) family (*HNRNPA2B1, HNRNPC* and *HNRNPG*), the m6A binding protein composed of insulin-like growth factor 2 mRNA binding proteins 1/2/3 (*IGF2BP1/2/3*) and eucaryotic initiation factor 3 (*eIF3*) [39,40] (Figure 1).

*YTHDF2* was the first RNA discovered to recognize and bind m6A. Its transcript degrades m6A modification by directly recruiting the carbon catabolite repression 4-negative on TATA-less (*CCR4-NOT*) deadenylase complex [41,42]. The three *YTHDF* proteins participate in similar biological pathways [43]. Recent investigations have shown that *YTHDF3* promotes the translation of methylated RNA by increasing the rate of protein synthesis in tandem with *YTHDF1*, and affects mRNA attenuation or translation mediated by *YTHDF2* through an interaction with *YTHDF2* [44,45]. Unlike *YTHDF2*, *IGF2BP*s identify m6A alteration under normal and stress conditions to alter gene regulation and tumor biology via their K homologous domains. To prevent m6A mRNAs from being degraded and to boost mRNA stability and translation, *IGF2BP*s interacts with the mRNA stabilizer HuR [46].

The *YTHDC1* is a unique nuclear m6A reader protein which has recognition sites in the nucleus. *YTHDC1* can interact with initiation factors and ribosomes, including *eIF3/4E/4G*, poly (A) binding protein (*PABP*), and 40S ribosomal subunits, to improve the translation efficiency of target RNA [47]. It can also regulate mRNA cleavage by coordinating and regulating pre-mRNA splicing factors, such as arginine-rich SR splicing factors (*SRSFs*) [48]. For example, by interacting with SRSF3, *YTHDC1* facilitates the nuclear export of m6A methylated mRNA in concert with nuclear RNA export factor 1 (*NXF1*) and the three prime repair exonuclease (*TREX*) mRNA export complex [49,50]. In addition to interacting with *YTHDC1*, *YTHDC2* can also bind to the 5’-3’ exoribonuclease 1 (*XRN1*) and meiosis-specific coiled-coil domain-containing protein (*MEIOC*) to increase target mRNA translation efficiency and decrease its expression [51,52]. Through its C-terminal region, certain m6A sites can also be located. To directly attract *CCR4–NOT* alkenylase complexes and transport RNA to the processor, the N-terminal portion of *CCR4–NOT* transfer complex subunit 1 (*CNOT1*) interacts with the Src homology domain. This promotes the rapid degradation of m6A-modified RNA. [41].

Members of the *HNRNP* family are important m6A readers. *HNRNPA2/B1* can selectively detect changes in m6A on transcripts and trigger various splicing events. Studies have demonstrated that *HNRNPA2/B1* can interact with drosha ribonuclease III (*DROSHA*) and the DiGeorge syndrome critical region 8 (*DGCR8*) to recognize m6A on a subset of primary miRNA transcripts, boosting primary miRNA processing [53]. Therefore, *HNRNPA2/B1*, which is a nuclear m6A binding protein, can affect the synthesis of miRNA [54]. In addition, m6A modification alters the RNA secondary structure. *HNRNPC* is a rich nuclear RNA binding protein that identifies the m6A-modified group, participates in pre-mRNA processing, and monitors the abundance of target transcripts and their alternative splicing [55]. By altering the m6A-modified RNA transcript, *HNRNPG* can induce similar effects to those of *HNRNPC*. In addition, it is known as the “m6A switch” because it controls the expression level of mRNA and its splicing mechanism [56].

## 3. m6A Modification and Cancer

### 3.1. Thyroid Carcinoma

**Figure 2:**
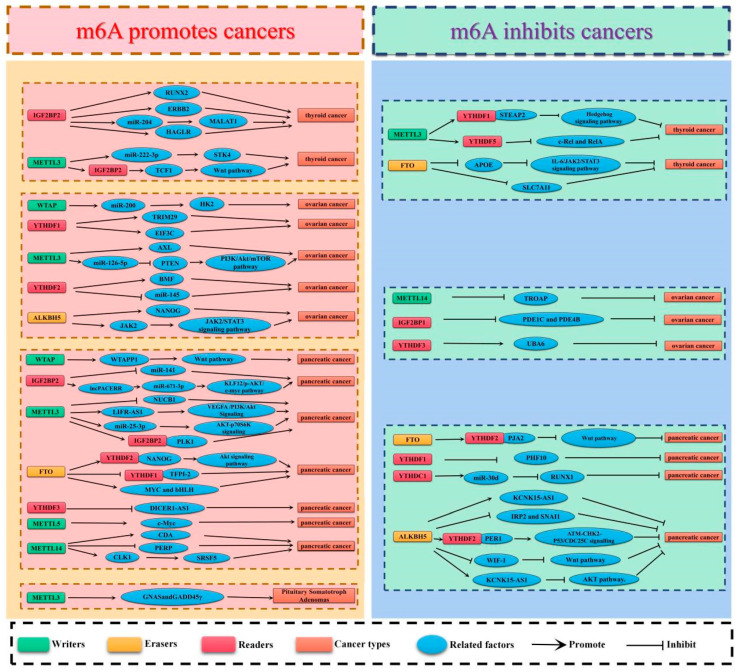
The potential roles of m6A in endocrine cancer progression. The potential role of m6A in endocrine cancer progression is reflected in the regulation of tumor-associated gene expression. m6A modification promotes cancer progression by enhancing oncogene expression and inhibiting tumor suppressor gene expression. m6A modification hinders cancer progression by inhibiting oncogene expression and enhancing tumor suppressor gene expression.

Thyroid carcinoma (TC) is the most prevalent endocrine-related malignancy. [57]. It is also the sixth most prevalent type of malignant tumor in the world, according to the 2020 Global Cancer Statistics [58]. The incidence rate increases by about 4% annually, accounting for 1% of all new cancers. Papillary thyroid carcinoma (PTC) is the main pathological subtype of TC [59], accounting for about 80–90% of all TC cases [60,61,62]. Most patients with TC have a good prognosis. Although PTC is well-differentiated, its biological performance is quite different. The inert thyroid micropapillary carcinoma shows a slow progression and rarely invades other tissues, whereas PTC shows lymph node or distant metastasis and has high mortality rates [63]. Recurrence and distant metastasis occur in about 30% of individuals, which negatively impacts patient prognosis [64,65]. Similar to other malignant tumors, the occurrence of TC is affected by environmental and genetic factors [66,67]. However, the exact molecular mechanism underlying the prevalence of TC remains elusive. Thus, understanding the role of m6A in TC is important to determine the most effective therapeutic strategy for TC (Table 1, Figure 2).

The function of *METTL3* in PTC is not well understood. Zhu et al. [68] reported that the oncogene *METTL3* can inhibit tumor growth, metastasis, and the invasiveness of tumors. It enhanced the expression and translation of six-transmembrane epithelial antigen of the prostate 2 (*STEAP2*) mRNA mainly by recruiting the m6A “interpreter“ protein *YTHDF1*. Aberrant stimulation of the Hedgehog signaling pathway and epithelial-mesenchymal transition (*EMT*) were linked to decreased *STEAP2* expression. *EMT* is a process through which cancer cells lose their epithelial properties and acquire stem cell-like invasive qualities which increases their capacity for local invasion and metastasis [114,115]. He et al. [69] found that *c-RelA* and *Rel1* are m6A targets downstream of *METTL3*, and *METTL3* can recognize *c-Rel* and *RelA* m6A modifications and cooperate with *YTHDF2* to regulate the consistency of *c-Rel1* and *RelA* mRNA, thereby triggering the nuclear factor kappa B (*NF-κB*) signaling pathway. In the chemotaxis test and mouse model experiments, it was found that an increased concentration of Interleukin-8 (*IL-8*) promoted the recruitment of tumor-associated neutrophils (*TANs*). Moreover, *METTL3* deficiency increased the production of *IL-8* in PTC cells, enhanced the recruitment of *TAN*, and promoted PTC progress. However, high *METTL3* expression was associated with a worse prognosis in patients with TC in some studies. [70,71]. For example, Wang et al. [70] reported that increasing *METTL3* expression promoted the migratory capacity and Wnt activity in TC cells through the regulation of m6A methylation on T cell factor 1 (*TCF1*). Moreover, the RIP assay showed that *TCF1* interacted with *METTL3* or *IGF2BP2*, and *METTL3* positively regulated the abundance of *TCF1*, thereby decreasing *IGFBP2* expression and accelerating the malignant behavior of the TC cell. Lin et al. [71] found that *METTL3* mediated the m6A modification of pre-miR-222-3p to enhance the maturation of pre-miR-222-3p and increase miR-222-3p expression. PTC tumor suppressor serine/threonine Stress kinase 4 (*STK4*) is a target of miR-222-3p, which has an anti-regulatory effect on *STK4*. Thus, *METTL3* knockdown can inhibit TC cell growth and malignant behavior.

Rescheduling energy metabolism through the activation of metabolic pathways such as glycolysis is a sign of cancer. Even in an aerobic environment, cancer cells revert to glycolysis for glucose metabolism rather than mitochondrial oxidative phosphorylation [116]. Huang et al. [72] postulated that PTC can be prevented by the activity of *FTO*, inhibiting the tumor development gene. Experimental studies showed that *FTO* inhibited the expression of Apolipoprotein E (*APOE*) through *IGF2BP2*-mediated m6A modification epigenetics. Furthermore, by modulating the *IL-6/JAK2/STAT3* signal pathway, *FTO* and *APOE* may limit the glycolysis of PTC, which could ultimately affect the growth of the tumor. The toxic buildup of lipid-based reactive oxygen species (ROS) causes ferroptosis, an iron-dependent form of regulated cell death. Abnormal levels of iron can cause various diseases. Ji et al. [73] performed MeRIP-seq and RNA-seq analyses and found that *FTO* regulates PTC cell ferroptosis and tumor progression by regulating the epigenetic activation of the *SLC7A11* gene.

Radioiodine (131-I) treatment can keep tumors in a “tumor dormancy” state for a long time, or even forever. It has few side effects and is a first-line treatment for a 131-I-avid disease with local recurrence and/or distant metastasis. However, if the dedifferentiation of PTC changes the mRNA or protein level of the iodine de-processing gene and loses the ability of iodine uptake and organization, then the tumor will not tolerate 131-I treatment. Radioactive iodine-resistant PTC is also referred to as radioactive iodine-refractory papillary thyroid carcinoma (RR-PTC) [117]. Clinically, the prognosis of RR-PTC is poor and researchers are attempting to develop effective interventions to improve patient outcomes. Some studies have proved that the m6A reader *IGF2BP2* can be used as a post-transcriptional regulator to participate in the differentiation of cancer and cancer stem cells [118], such as hepatocellular carcinoma [119], liposarcoma [120], and glioblastoma [121]. *IGF2BP2*-dependent activation of the Erb-B2 receptor tyrosine kinase 2 (*ERBB2*) signal is the cause of acquired tyrosine kinase inhibitor (TKI) resistance. PTC cells can become resistant to TKIs because *IGF2BP2* increases the expression of the *ERBB2* protein by binding to the m6A methylation site of *ERBB2* mRNA and thereby promoting the expression of *ERBB2*. This in turn increases the constitutive activation of signal pathways involved in the dedifferentiation process of PTC [74]. Another study by Sa et al. [75] found that metastatic PTC with high expression of *IGF2BP2* was prone to 131-I incompatibility, and *IGF2BP2* could integrate with 30-UTR of runt-related transcription factor 2 (*RUNX2*), thus damaging the stability of *RUNX2* mRNA. *RUNX2*, as a transcription factor, can combine with the promoter of iodide binding gene *NIS* to regulate its activity. *RUNX2* can inhibit the expression of *NIS* and then cause the differentiation of RR-PTC. Therefore, decreasing *IGF2BP2* and increasing *RUNX2* mRNA expression can restore the iodine uptake capacity of RR-PTC. In addition to overcoming the acquired drug resistance to RR-PTC, there is evidence from a few studies that *IGF2BP2* is also strongly expressed in TC, which has been proven to be the target of miR-204. It can competitively bind miR-204 with metastasis-associated lung carcinoma transcript one, recognize and upregulate *IGF2BP2* through m6A modification, and enhance myelocytomatosis oncogene (*MYC*) expression, thus stimulating the proliferation, migration, and invasion of TC cells [76]. Dong et al. [77] found that *IGF2BP2* can also induce TC cell apoptosis by downregulating lncRNA-*HAGLR* and inhibiting TC cell proliferation, cell cycle progression, cell migration, and invasion.

### 3.2. Ovarian Cancer

For women, ovarian cancer (OC) has the highest fatality rate of any gynecological malignancy [122]. It is the fifth leading cause of cancer-related deaths among women worldwide [123,124], with a 5-year survival rate of less than 45% [122]. About half of OC patients are diagnosed at an advanced stage, which contributes to the poor overall survival rate [125,126]. OC is divided into three common types, including epithelial ovarian cancer (EOC), germ cell tumors, and sex cord-stromal tumors [127]. EOC accounts for more than 90% of all cases of OC [128]. Although surgery and targeted chemotherapy have made progress [129], the prognosis for most EOC patients is poor because of their inherent high recurrence rate and resistance to cisplatin [122]. Accumulating evidence shows that OC has a high degree of heterogeneity, which leads to the continuous growth and recurrence of chemotherapy-resistant cancer cells [130]. In addition, the primary cause of death in people with OC is tumor growth and spread, and there is currently no medication that can stop this from happening [131,132]. Here, we explore the role of m6A in regulating the development of OC through modulating protein translation. (Table 1, Figure 2).

Bi et al. [78] found that through m6A modification of pri-miR-126-5p, *METTL3* stimulates miR-126-5P maturation. It was confirmed that miR-126-5p could activate the *PI3K/Akt/mTOR* pathway by directly targeting phosphatase and tensin homolog (*PTEN*). Inhibition of *METTL3* via miR-126-5p knockdown slows the progression and carcinogenesis of OC by blocking the activation of the *PI3K/Akt/mTOR* pathway. Hua et al. [79] used a nude mouse model to show that steady overexpression of *METTL3* significantly enhanced cell proliferation, lesion development, motility, and invasion in nude mice. *METTL3* has also been found to promote EMT by up-regulating receiver tyrosine kinase *AXL*. Multiple studies have linked EMT to OC cell invasion and metastasis [133,134,135]. By promoting *AXL* translation and EMT, it is assumed that *METTL3* plays a crucial carcinogenic role in the formation and/or invasion of OC. In addition to *METTL3*, Li et al. [80] found that in contrast to normal tissues, OC tissues have significantly reduced levels of *METTL14* and m6A RNA methylation. The decreased expression of *METTL14* was also associated with the change in the copy number variations (CNVs) and the low survival rate of OC patients. As a negative regulator, *METTL14* can regulate the stability of the cell-proliferative gene troppin-associated protein (*TROAP*) mRNA and then prevent the growth of OC cells.

*WTAP* plays a crucial role as a “writer” in m6A modification. As a nuclear protein involved in cell function and cancer progression, it is frequently found close to splicing factors. Yet, *WTAP*’s role and mechanism in OC are largely mysterious. Lyu et al. [81] discovered that the hypoxic microenvironment influences the glycolysis pathway of cancer cells, hence driving the biological process of OC growth. They also, for the first time, reported the hypoxia-inducible factor-1α (*HIF-1α*). *HIF-1α* can actively regulate the increase in *WTAP* expression under hypoxia. A low survival rate is associated with high *WTAP* expression in OC patients. miR-200 expression in OC cells is regulated by *WTAP* via interactions with *DGCR8*, a component of the usual microprocessor complex of miRNA synthesis. miR-200 can positively regulate the key glycolytic enzyme hexokinase 2, significantly influence the intracellular Warburg effect, and promote tumor events and progress.

In addition, Nie et al. [82] discovered that upregulation of *ALKBH5* in EOCs triggered resistance to cisplatin. Further analysis showed that homeobox A10 (*HOXA10*) is a transcription factor that promotes *ALKBH5* transcription, and *ALKBH5* can also control its activity. As a m6A-modified gene, Janus kinase 2 (*JAK2*) is one of *ALKBH5*’s primary targets. *ALKBH5* maintains the stability of *JAK2* mRNA in a *YTHDF2*-mediated manner. By persistently stimulating the signaling pathway of *JAK2/STAT3*, which is m6A-dependent, upregulation of the *ALKBH5-HOXA10* loop enhances EOC tumor development and cisplatin resistance. To avoid cisplatin resistance in EOC, it may be possible to employ the technique of suppressing the expression of the *ALKBH5-HOXA10* loop. Jiang et al. [83] reported higher levels of *ALKBH5* expression in OC tissues compared with normal ovarian tissues, but the *ALKBH5* expression levels were decreased in OC cell lines. Toll-like receptor 4 (*TLR4*), a molecule that participates in the tumor microenvironment (TME), showed a similar expression trend. Jiang et al. set up a macrophage and OC cell co-culture paradigm in vitro to examine the impact of aberrant TME on OC progression. An increased expression of *ALKBH5* and *TLR4* was observed in OC cells co-cultured with M2 macrophages, suggesting that *TLR4* activates *NF-κB*. After mRNA demethylation, the expression of *NANOG* increased, which promoted the invasiveness of OC cells.

Huang et al. [84] found that tumorigenesis and cancer stem cell (CSC) self-renewal in ovarian cancer were both suppressed by *FTO* treatment, which is the first study to link *FTO* to the cell regulatory process mediated by the second messenger cyclic adenosine monophosphate (cAMP). The study showed that compared with non-CSCs, the cAMP level in ovarian CSCs was significantly decreased, and *FTO* overexpression induced an increase in the cAMP content, while its knockdown reduced the cAMP level. In addition, by reducing the m6A level at the 3′UTR and the mRNA stability of phosphodiesterase genes (*PDE1C and PDE4B*), *FTO* enhanced the cAMP signal and inhibited the stem cell characteristics of OC cells. In summary, this finding demonstrates that the cAMP pathway is a crucial *FTO* target in CSCs and reveals important insights for developing improved therapies for high-grade malignant ovarian cancer.

Due to the aggressive nature of EOC, chemotherapy resistance continues to be a major contributor to poor prognosis [85]. In cisplatin-resistant OC cells and clinical tissues, Hao et al. [86] found that tripartite motif-containing protein 29 (*TRIM29*) can enhance CSC-like characteristics of cisplatin-resistant OC cells as an oncogene. Moreover, *YTHDF1* knockdown can inhibit the CSC-like characteristics of cisplatin-resistant OC cells and save this inhibition by increasing the expression of *TRIM29*. It is proved that *YTHDF1*, as an upstream molecule of *TRIM29*, can be recruited by *TRIM29* to recognize its 3′UTR, participate in m6A modification, and promote its translation in cisplatin-resistant OC cells in an m6A-*YTHDF*-dependent manner.

A tumor suppressor factor named F-box and WD repeat domain-containing 7 (*FBW7*) recognizes substrates as part of the Skp1-cullin 1-F-box (*SCF*) E3 ubiquitin ligase complex, which catalyzes the degradation of many cancer-causing proteins. Xu et al. [87] found that compared with normal ovarian tissues, the expression of *FBW7* was significantly downregulated in EOC tissues, whereas the expression of *YTHDF2* was significantly elevated. *YTHDF2* was identified as a new substrate of *FBW7*. By stimulating the proteasome degradation of *YTHDF2* in OC, *FBW7* mitigates *YTHDF2*’s tumor-promoting impact. In addition, *YTHDF2* regulates the turnover of the m6A-modified mRNA apoptosis promoting gene Bcl2 modifying factor (*BMF*). It has been shown that by inhibiting *YTHDF2*-mediated *BMF* mRNA downregulation, *FBW7* inhibits tumor growth and metastasis in OC. In addition to being identified as a new substrate of *FBW7*, *YTHDF2* is also a target gene of miR-154. In OC, there is an inverse relationship between miR-145 and *YTHDF2* expression levels. Overexpression of *YTHDF2* has been shown to rescue miR-145-induced reductions in EOC proliferation and migration. Therefore, through m6A alteration, *YTHDF2* and miR-145 can create a negative feedback mechanism that controls OC growth [88].

Wang et al. [89] demonstrated that lncRNA the ubiquitin-like modifier activating the enzyme 6 antisense RNA 1 (*UBA6-AS1*) was significantly associated with the prognosis of OC patients and was involved in the regulation of m6A. Positive correlations were also seen between *UBA6-AS1* and *UBA6* mRNA expression in OC tissues, and *UBA6-AS1* was reported to prevent the degradation of *UBA6* mRNA. By interacting with *UBA6*, *UBA6-AS1* reduces the proliferation, migration, and invasion of OC cells. The reason for the positive correlation is that *UBA6-AS1* increases the m6A methylation of *UBA6* mRNA through *RBM15*. *UBA6-AS1-RBM15*-mediated m6A modification of *UBA6* mRNA improved *UBA6* mRNA stability, and *IGF2BP1* was identified as the m6A reader protein.

### 3.3. Pancreatic Cancer

In terms of cancer-related mortality, pancreatic cancer (PC) is in the top seven of the worst diseases. [136,137]. The 5-year survival rate for pancreatic cancer is only 8%, and lowers to 3% at the distant metastatic stage [137]. Most patients have been diagnosed with unresectable local advanced or metastatic diseases because of the late onset of PC symptoms. Metastasis, recurrence, and chemotherapy resistance may lead to adverse outcomes. Over 90% of pancreatic illnesses can be attributed to pancreatic duct adenocarcinoma (PDAC), making it the most common form of PC [138,139]. The clinical prognosis of PDAC is poor [140]. Approximately 10% of people diagnosed will survive for 5 years after diagnosis [141,142]. Despite the fact that few patients were diagnosed with resectable local tumors, the 5-year survival rate following surgery was just 20% [143]. Therefore, m6a’s pathophysiological role and expression profile in PC must be determined (Table 1, Figure 2).

Through hypertranscriptional analysis, Tatekawa et al. [90] identified the polo-like kinase 1 (*PLK1*) gene in patients with PC which affects the prognosis. They found that *METTL3* regulates the cell cycle of PC cells through the methylation of the 3′UTR of *PLK1*, which disrupts homeostastic balance and increases cell death by increasing replication stress. The study also found that *IGF2BP2* binds to m6A in the 3’UTRs of *PLK1*, thereby increasing its expression. Demethylation of this region results in ataxia, telangiectasia, and increased radiosensitivity, which activates the Rad3-related protein pathway by elevating replication stress and formation of mitotic mutations. This demonstrates the critical role of *PLK1* methylation in the PC cell cycle and shows its potential to be a new radiation and sensitization treatment target.

In addition, Hua et al. [91] noted that the *METTL3* induced the m6A modification of Nucleobindin 1 (*NUCB1*) 5′UTR through the *YTHDF2* reader. *NUCB1* showed an additive effect with gemcitabine in vitro and in vivo. A decrease in PC cell proliferation was seen after *NUCB1* overexpression. *NUCB1* further inhibited the gemcitabine-induced unfolded protein response and autophagy by controlling activating transcription factor 6 activity and enhancing the effect of gemcitabine. Zhang et al. [92] reported the carcinogenic effect of miR-25-3p generated by cigarette smoke condensate via the m6A pathway in pancreatic cells. Cigarette smoke condensate can promote the excessive maturation of carcinogenic primary microRNA-25 (pri-miR-25) in pancreatic ductal epithelial cells through NF-kappaB-associated protein (*NKAP*)-mediated increased m6A modification. Because cigarette smoke condensate also causes hypomethylation of the *METTL3* promoter, increased expression of this enzyme is required to catalyze this change. In terms of the splice site for pri-miR-25, the RNA-binding protein *NKAP*, as an m6A reader, preferentially binds to the common motif RGm6AC. By promoting the interaction between pri-miR-25 and the protein *DGCR8* of the miRNA microprocessor complex, it enhances the maturation of miR-25-3P. Overexpression of miR-25-3p targets the pleckstrin homology domain and leucine-rich repeat protein phosphatase 2 (*pHLPP2*) mRNA and significantly inhibits its expression. This in turn stimulates *AKT-p70S6K* signal transduction. Therefore, the *METTL3/miR –25–3p/pHLPP2/AKT* axis plays a role in the initiation and progression of PDAC. Chen et al. [93] pointed out that *METTL3* induced m6A methylation on the 3′UTR of leukemia inhibitory factor receptor antisense RNA 1 (*LIFR-AS1*) to enhance its mRNA stability, and also determined that *LIFR-AS1* expression increased in PC cell lines and tumors. Through direct interaction with miR-150-5p, *LIFR-AS1* increases expression of vascular endothelial growth factor A (*VEGFA*). It was found that *LIFR-AS1* knockdown had adverse effects on the *VEGFA/PI3K/Akt* signal, which was reversed through the suppression of miR-150-5p expression. It is demonstrated that by regulating miR-150-5p/*VEGFA*, the novel *METTL3/LIFR–AS1* axis stimulates PC development.

A pioneering study by Huang et al. [94] showed that the oncogene *METTL5* promoted the proliferation, motility, invasion, and tumorigenesis of PC cells. Enhanced *c-Myc* translation may contribute to the carcinogenic role of *METTL5*. *METTL5* is specifically regulated during translation thanks to the 5’untranslated region (5’UTR) of *c-Myc* mRNA and the m6A alteration of the coding DNA sequence region (near the 5’UTR). *METTL5* and its cofactor *TRMT112* synergistically promote the progression of PC.

Gemcitabine treatment dramatically upregulated m6A methyltransferase *METTL14* expression in pancreatic cancer patients, as reported by Zhang et al. [95]. In addition, the reduction in *METTL14* expression increased the sensitivity of drug-resistant cells to gemcitabine therapy. Mechanistically, they discovered that the transcription factor p65 can up-regulate the production of *METTL14* by binding to its promoter region, which in turn increases the expression of the enzyme Cytidine deaminase (*CDA*), which inactivates gemcitabine. These findings suggest that reducing patient drug resistance by targeting *METTL14* may be possible. Wang et al. [96] found that *METTL14* overexpression directly targeted the downstream *PERP* mRNA (p53 effector related to PMP-22), which increased the turnover of *PERP* mRNA and reduced *PERP* expression in PC cells. There is evidence that *PERP* has a role in maintaining the integrity and homeostasis of epithelial cells, and that it regulates the adhesion subprogram (which influences cell death) [144]. In this way, it dramatically stimulates PC cells’ growth and migration. In addition, it participates in DNA damage-induced apoptosis through a mechanism dependent or independent of the *p53* signaling pathway [145]. In a study on changes in the alternative splicing mode of m6A modification-related proteins in PC, Chen et al. [97] found that the alternative splicing mode of *METTL14* regulates the process of m6A methylation. Another study showed that *CLK1* altered the alternative splicing mode of *METTL14* by activating SRSF5, promoted cyclin L2exon6.3 jumping, and inhibited *METTL14* exon10 jumping, which accelerated cell proliferation, migration, and invasion.

Liu et al. [98] discovered that LncRNA-*PACERR* was increased in PC leading to enhanced tumor development, and LncRNA-*PACERR* promoted pancreatic cancer development by acting on *IGF2BP2*. *PACERR* collaborated with *IGF2BP2* to increase *KLF12* and c-myc expression. *KLF12* and c-myc are both transcription factors; however, their precise regulatory locations are different. To stabilize *KLF12* and c-myc, the *PACERR* interacts with a region on the secondary structure of the *IGF2BP2* protein, which is located close to the *KH1* and *KH2* domains, in an m6A-dependent manner. By binding to miR-671-3p, the *PACERR* contributes to the occurrence of PDAC by activating the *KLF12/p-AKT/c-myc* signaling pathway. In addition, *KLF12* specifically targets the promoter of LncRNA-*PACERR*. LncRNA-*PACERR* increases histone acetylation and promotes PC development through interactions with *KLF12* in the nucleus when *EP300* is recruited. Xu et al. [99] confirmed that the miR-141 genome amplification and silencing resulted in *IGF2BP2* activation. Given that *IGF2BP2* is a direct target of miR-141, the above finding shows a negative association between them. In addition, it was reported that an increased expression of *IGF2BP2* stimulated the proliferation of PC cells via the *PI3K/Akt* signaling pathway.

Wang et al. [100] showed that *FTO* knockdown increased the methylation of *TFPI-2* mRNA in PC cells, and the m6A reader *YTHDF1* increased the stability of *TFPI-2* mRNA, leading to the upregulation of *TFPI-2* expression. This inhibited PC cell proliferation, colony formation, spheroid formation, migration, and invasion. Tang et al. [101] also observed reduced proliferation and DNA synthesis in PC cells following suppression of the *FTO* gene. Research has shown that *FTO* can decrease the quantity of the m6A protein, which stabilizes the *MYC* proto-oncogene *bHLH* transcription factor. This upregulates *bHLH* transcription factor expression, thereby reducing cell apoptosis and promoting tumor proliferation and growth. Tan et al. [102] found that *FTO* directly targeted the platelet-derived growth factor C (*PDGFC*), resulting in the formation of PDGFC 3ʹ in UTR, m6A modification was reduced and its mRNA expression was stabilized in an m6A-YTHDF2 dependent manner while the expression of PDGFC was increased. Consequently, the Akt signaling pathway was reactivated, which promoted cell growth, colony formation, and tumor progression. Zeng et al. [103] also found that *FTO* modified and demethylated m6A of the praja ring finger ubiquitin ligase 2 (*PJA2*) in an m6A-*YTHDF2* dependent manner, enhancing the stability of *PJA2* and stabilizing its mRNA and inhibiting the Wnt signaling pathway. In conclusion, these complex mechanisms regulate the proliferation, invasion, and metastasis of PC cells.

A study by Huang et al. [104] revealed a new DNA damage response mechanism involving the m6a modification of the chromatin remodeling complex subunit *PHF10*. They discovered that via increasing m6A alteration in PDAC cells, fluoxetine causes DSB and inhibits HR repair. The *PBAF* chromatin remodeling complex, which is thought to be the primary molecule impacted by non-sit-in therapy, contains the m6A writers *ZC3H13* and *PHF10*. The *ZC3H13*-mediated m6A RNA modification inhibits *PHF10* translation, allowing its participation in DNA damage response processes. The loss of *PHF10* function increases the possibility of *H2AX, RAD51,* and *53BP1* recruitment to DSB sites, and the effectiveness of HR repair is reduced. Additionally, ZC3H13 regulates PHF10 translation and m6A methylation in a *YTHDF1*-dependent way. This discovery contributes to our knowledge of the mechanism of DNA repair and suggests potential treatments for PC.

Hu et al. [105] found that *DICER1-AS1* induced transcription of its sense gene by promoting binding of the transcription factor *YY1* to the *DICER1* promoter. In addition, *DICER1* enhanced the maturation of miR-5586-5p leading to the inhibition of the expression of glycolytic genes including *LDHA, HK2, PGK1*, and *SLC2A1*. These findings demonstrate that *YTHDF3* is a key target of miR-5586-5p that regulates PC glycolysis by forming a negative feedback loop with *DICER1-AS1*. Glucose depletion enhances the interaction between *YTHDF3* and *DICER1-AS1* and glucose deprivation, leading to the degradation of *DICER1-AS1*.

Deng et al. [106] found that the mRNA of *WTAPP1* was overexpressed in patients with PDAC, which correlated with poor survival. m6A modification of CCHC type zinc filament nuclear acid binding protein (*CNBP*) was reported to stabilize *WTAPP1* RNA, thereby increasing the *WTAPP1* RNA expression level in PDAC cells. The overexpressed *WTAPP1* RNA can bind to its protein-coding counterpart *WTAP* mRNA, and several *EIF3* translation initiation complexes are recruited to promote *WTAP* translation. The increase in *WTAP* protein levels in turn enhances the activation of the *Wnt* signaling pathway and induces the malignant phenotype of PDAC.

Hou et al. [107] found that *YTHDC1* promotes the biosynthesis of mature miR-30d by regulating the m6A-mediated mRNA stability. Subsequently, miR-30d directly targets the transcription factor *RUNX1*, which binds to the promoters of the *SLC2A1* gene and *HK1* gene to inhibit aerobic glycolysis. The amount of m6A modified pri-miR-30d was dramatically decreased by the deletion of *METTL3*/*14*. To start miR-30d biosynthesis, *YTHDC1* antagonizes the termination of *MCPIP1*’s miRNA biogenesis by preferentially recognizing m6A-modified pri-miR-30d and promoting its degradation. The axis of *YTHDC1-miR-30-RUNX1-SLC2A1/HK1* is created.

Guo et al. [108] found that *ALKBH5* activated *PER1* by inducing m6A demethylation and transcription in an m6A-*YTHDF2*-dependent manner. The upregulation of *PER1* triggered a reactivation of the *ATM-CHK2-P53/CDC25C* signaling pathway which inhibits tumor proliferation, migration, and invasion. *PER1*-induced *P53* upregulation and *P53*-activated *ALKBH5* transcription are important indicators of the feedback regulation of m6A modification in PC, creating an *ALKBH5-PER1-P53-ALKBH5* feedback loop. Studies have demonstrated that *ALKBH5* demethylates *KCNK15-AS1* and participates in *KCNK15-AS1*-mediated cell migration and invasion, hence regulating tumor growth [109,110]. In a pioneering study by He et al. [109], it was confirmed that *KCNK15-AS1* inhibits PC cell growth by regulating *KCNK15* and *PTEN*. It was also found that *ALKBH5*-mediated m6A demethylation enhanced the stability of *KCNK-AS1*. The *KCNK15-AS1* binds to *KCNK15* 5’UTR, leading to the inhibition of *KCNK15* translation. In addition, *KCNK15-AS1* recruits the *MDM2* proto-oncogene (*MDM2*) and interacts with it to promote the ubiquitination of the RE1 silencing transfer factor (*REST*), transcriptionally upregulating phosphate and tensin homolog (*PTEN*) and thus inactivating the *AKT* pathway [110]. Huang et al. [111] revealed that *ALKBH5* protects PDAC by regulating iron metabolic regulators. They also reported that the mRNA encoding ubiquitin ligase *FBXL5* and mitochondrial iron introns *SLC25A28* and *SLC25A37* are potential substrates of *ALKBH5* and that their stability is regulated by *ALKBH5*. The *ALKBH5* overexpression that was triggered by *FBXL5*-mediated degradation led to a dramatic reduction in the levels of the iron-regulatory protein *IRP2* and the EMT regulator *SNAI1*. Therefore, cellular iron levels and migratory and invasive capacities were significantly decreased. Tang et al. [112] showed that gemcitabine inhibited *ALKBH5* expression in a patient-derived xenograft (*PDX*) model. The experiment proved that *ALKBH5* deficiency promoted the proliferation, migration, and invasion of PDAC cells in vitro and in vivo, and its overexpression increased the sensitivity of PDAC cells to chemotherapy. Moreover, the m6A expression profile of some *ALKBH5* target genes was altered, including that of Wnt aggression factor 1 (*WIF-1*).

## 4. Pituitary Adenoma

Near the base of the brain is a small gland called the pituitary, which is usually called the “main gland”. It is the most significant endocrine gland in the body, regulating the secretion of essential hormones. These hormones regulate vital bodily processes, including growth, blood pressure, reproduction, and metabolism [146,147]. 10–20% of all brain cancers are pituitary adenomas (PA), making them the second most prevalent type of brain tumor [148,149]. Previously, PA was classified according to its size. Those less than 10 mm were named microadenomas, and the rest were referred to as macroadenomas [150]. Currently, it is classified according to the type of hormone it excessively secretes into the blood, e.g., adrenocorticotropic hormone-secreting adenoma, growth hormone-secreting adenoma, prolactin-secreting adenoma, and thyroid hormone-secreting adenoma [151]. It is estimated that 35% of pituitary adenomas will show infiltration of the cavernous sinus and sphenoid sinus [152]. Transnasal transsphenoidal surgery is the main treatment for PAs [153]. In clinical practice, PAs that infiltrate the suprasellar or parasellar regions are hard to eradicate completely, and patients with a residual adenoma have a recurrence rate of 12–58% [149]. For instance, 10–20% of the adenoma will recur within 5–10 years, even when the adenoma is completely removed [154]. To achieve total tumor control or biochemical remission, drugs and stereotactic radiosurgery are suggested. [155,156]. Therefore, identification of drug targets through m6A modification research may generate strategies that will form the basis for clinical treatment (Table 1, Figure 2).

In a study of endocrine pituitary adenomas, it was found that the expression of *METTL3* messenger RNA and protein in GH-PA samples was higher than that in normal pituitary tissue samples and non-secretory pituitary adenomas. The level of m6A modification in GH-PA was increased, and hypermethylated RNA was involved in hormone secretion and cell development. In GH3 cell lines, the absence of *METTL3* decreased cell proliferation and GH secretion. They discovered that *GNAS* and *GADD45* act as targets downstream of this mechanism. In addition, m6A methyltransferase *METTL3* increased *GNAS* and *GADD45* in an m6A-dependent manner, which promoted tumor growth and hormone secretion. These findings demonstrate that *METTL3* and methylated RNA are potential targets for the clinical treatment of GH PAs [113].

## 5. The Future Perspectives on the Application of Targeted m6A Therapy in the Treatment of Endocrine Cancer

It should be noted that m6A modification is one kind of epigenetic modification to RNA. Studies into the mechanism of m6A alteration have revealed that it adds a layer of complexity to the regulation of RNA levels. The current hypothesis on the link between m6A and cancers is that m6A is neither good nor bad. It can either promote or inhibit cancer cell growth by affecting the mRNA level of related oncogenes or suppressor genes. The degree of m6A modification of RNA correlates closely with the level of expression of writing and clearing genes within cells. Many biological processes are initiated by attachment of reader proteins to the m6A modification site. In general, an aberrant expression of m6A may disrupt the normal RNA modification process and directly interfere with the processing, transport, translation, and destruction of mRNA, which may contribute to the development and occurrence of malignancies. In addition, besides m6a-modified mRNA, the role of m6a-modified non-coding RNA in endocrine cancer should be explored. A gene’s expression level can be modulated by ncRNA. The m6A alteration of ncRNA impacts its own expression level. The bidirectional regulation of m6A modification, particularly the regulation of ncRNA, may provide a new explanation for certain disorders. Mutations at the m6A site may disrupt the regulation of the m6A factor, especially “author” and “erasure”, leading to abnormal m6A modification levels in significant transcripts which is essential to the abnormal expression of related oncogenes. This calls for research focus on the mutation of the m6A locus, in various aspects such as single nucleotide polymorphism, which will improve the targeting of endocrine cancer. Based on their catalytic activity, numerous RNA-modifying enzymes have been shown to play key roles in the occurrence or maintenance of various forms of cancer [157]. Several m6a-related genes and proteins in tumors have the potential to be used as diagnostic markers and as therapeutic targets. Epigenetic mechanisms targeting has emerged as a promising new therapeutic approach. Targeting the m6A RNA modification pathway may prevent the onset and metastasis of cancer [158,159]. The development of RNA-modifying enzyme inhibitors will pave the way for a significant and novel approach to the treatment of tumors and other diseases. Small molecule inhibitors that target effector proteins involved in RNA methylation may hold tremendous potential as disease treatments. It is anticipated that RNA epigenetic medications may be applied in clinical practice to treat diseases. Given the numerous types of non-coding RNA, further research is advocated to identify novel therapeutic targets. Combining targeted mRNA and non-coding RNA therapy may offer an effective treatment for the seemingly intractable problem of endocrine cancer.

## 6. Conclusions

Abnormal changes in levels of RNA m6A modification or m6A regulatory factors have an important impact on the occurrence, development, metastasis, and prognosis of endocrine tumors. We provide a theoretical foundation for the development of new diagnostic markers and therapeutic techniques based on m6A alteration and regulators, and we discuss the knowledge about the role of m6A in endocrine cancers and its regulators and mechanisms. The data reviewed here provide a reference for further investigations into the carcinogenic or antitumor mechanism of the m6A regulators which may lead to the development of specific agonists or inhibitors of protein targets.

## Figures and Tables

**Table 1:** The roles of m6A enzymes in endocrine cancer progression.

Endocrine Cancer	m6A Component	Function	Role in Disease	Regulation	Related Target	Mechanism	Year	Ref
Thyroid cancer	METTL3	Writer	Anti-oncogene	Upregulation	YTHDF1/STEAP2	METTL3 stabilized STEAP2 mRNA and regulated STEAP2 expression positively through YTHDF1-mediated m(6)A modification. METTL3–STEAP2 axis functions as an inhibitr in PTC by suppressing epithelial-mesenchymal transition and the Hedgehog signaling pathway.	2022	[68]
	METTL3	Writer	Anti-oncogene	Downregulation	YTHDF2/c-Rel and RelA	METTL3 played a pivotal tumor-suppressor role in PTC carcinogenesis through c-Rel and RelA inactivation of the nuclear factor κB (NF-κB) pathway by cooperating with YTHDF2 and altered TAN infiltration to regulate tumor growth	2021	[69]
	METTL3	Writer	Oncogene	Upregulation	IGF2BP2/TCF1	Upregulated m6A methyltransferase METTL3 recruits IGFBP2 and promotes the progression of thyroid carcinoma through m(6)A methylation on TCF1	2020	[70]
	METTL3	Writer	Oncogene	Upregulation	miR-222-3p/STK4	METTL3 stimulated miR-222-3p expression by mediating the m6A modification of pri-miR-222-3p. miR-222-3p targeted and inversely regulated serine/threonine stress kinase 4 (STK4), thereby increasing the malignant behaviors of TC cells	2022	[71]
	FTO	Eraser	Anti-oncogene	Downregulation	APOE	FTO inhibited expression of APOE through IGF2BP2-mediated m(6)A modification and may inhibit glycolytic metabolism in PTC by modulating IL-6/JAK2/STAT3 signaling pathway.	2022	[72]
	FTO	Eraser	Anti-oncogene	Downregulation	SLC7A11	FTO functions as a tumor suppressor gene in PTC and is able to inhibit the occurrence of PTC by downregulating SLC7A11 in m6A independently.	2022	[73]
	IGF2BP2	Reader	Oncogene	Upregulation	ERBB2	IGF2BP2 bound to the N6-methyladenosine-binding site in the coding sequence of ERBB2 mRNA, yielding an increased ERBB2 translation efficacy. Inhibition of ERBB2 and IGF2BP2 rescued the PTCSR cells from acquired dedifferentiation.	2022	[74]
	IGF2BP2	Reader	Oncogene	Upregulation	RUNX2	IGF2BP2 bound to the m6A modification site of runt-related transcription factor 2 (RUNX2) 3’-UTR and enhanced the RUNX2 mRNA stability. Moreover, RUNX2 could bind to the promoter region of NIS to block the differentiation of RR-PTC	2022	[75]
	IGF2BP2	Reader	Oncogene	Upregulation	miR-204/MALAT1	MALAT1 contributes to TC progression through the upregulation of IGF2BP2 by binding to miR-204.	2021	[76]
	IGF2BP2	Reader	Oncogene	Upregulation	HAGLR	IGF2BP2 loss inhibited cell proliferation, migration and invasion, and induced cell apoptosis and cell cycle arrest by down-regulating HAGLR expression in an m6A-dependent manner in TC cells	2021	[77]
Ovarian cancer	METTL3	Writer	Oncogene	Upregulation	miR-126-5p/PTEN	Knockdown of METTL3 inhibited the effect of miR-126-5p to upregulate PTEN, and thus prevents PI3K/Akt/mTOR pathway activation, thereby suppressing the development of ovarian cancer.	2021	[78]
	METTL3	Writer	Oncogene	Upregulation	AXL	METTL3 promoted EMT by upregulating the receptor tyrosine kinase AXL, thereby facilitating metastasis of ovarian cancer.	2018	[79]
	METTL14	Writer	Anti-oncogene	Downregulation	TROAP	METTL14 overexpression decreased ovarian cancer proliferation by inhibition of TROAP expression via an m(6)A RNA methylation-dependent mechanism.	2022	[80]
	WTAP	Writer	Oncogene	Upregulation	miR-200/HK2	HIF-1α could positively regulate increased expression of WTAP under hypoxia; WTAP interacts with DGCR8 (a crucial chip protein) to regulate the expression of miR-200 in an m6A-dependent way; key glycolysis enzyme HK2 could be positively regulated by miR-200, which significantly affected the intracellular Warburg effect.	2022	[81]
	ALKBH5	Eraser	Oncogene	Upregulation	HOXA10/JAK2	HOXA10 formed a loop with ALKBH5 and was found to be the upstream transcription factor of ALKBH5. JAK2 is the m6A-modified gene targeted by ALKBH5. The JAK2/STAT3 signaling pathway was activated by overexpression of the ALKBH5-HOXA10 loop, resulting in EOC chemoresistance.	2021	[82]
	ALKBH5	Eraser	Oncogene	Upregulation	NANOG	TLR4 up-regulated ALKBH5 expression via activating the NF-κB pathway. NANOG served as a target in ALKBH5-mediated m6A modification. NANOG expression increased after mRNA demethylation, consequently enhancing the aggressiveness of ovarian cancer cells.	2020	[83]
	FTO	Eraser	Anti-oncogene	Downregulation	PDE1C and PDE4B	By reducing m(6)A levels at the 3’UTR and the mRNA stability of PDE1C and PDE4B, FTO augmented second messenger 3’, 5’-cAMP signaling and suppressed stemness features of ovarian cancer cells.	2020	[84]
	YTHDF1	Reader	Oncogene	Upregulation	EIF3C	YTHDF1 augments the translation of EIF3C in an m6A-dependent manner by binding to m6A-modified EIF3C mRNA and concomitantly promotes the overall translational output, thereby facilitating tumorigenesis and metastasis of ovarian cancer.	2020	[85]
	YTHDF1	Reader	Oncogene	Upregulation	TRIM29	TRIM29 could act as an oncogene to enhance the CSC-like characteristics of the cisplatin-resistant ovarian cancer cells. In addition, recruitment of YTHDF1 to m6A-modified TRIM29 was involved in promoting TRIM29 translation in the cisplatin-resistant ovarian cancer cells.	2021	[86]
	YTHDF2	Reader	Oncogene	Upregulation	BMF	Ectopic FBW7 inhibits ovarian cancer cell survival and proliferation. FBW7 counteracts the tumor-promoting effect of YTHDF2 by inducing proteasomal degradation of the latter in ovarian cancer. Furthermore, YTHDF2 globally regulates the turnover of m(6)A-modified mRNAs, including the pro-apoptotic gene BMF.	2021	[87]
	YTHDF2	Reader	Oncogene	Downregulation	miR-145	YTHDF2 was the direct target gene of miR-145. A crucial crosstalk occurred between miR-145 and YTHDF2 via a double-negative feedback loop. YTHDF2 and miR-145 were involved in the progression of EOC by indirectly modulating m6A levels.	2020	[88]
	IGF2BP1	Reader	Anti-oncogene	Upregulation	UBA6	UBA6-AS1 increased the m6A methylation of UBA6 mRNA via recruiting RBM15. IGF2BP1 as the m6A reader protein enhanced the stability of UBA6 mRNA.	2022	[89]
pancreatic cancer	METTL3	Writer	Anti-oncogene	Upregulation	IGF2BP2/PLK1	IGF2BP2 binds to m6A of PLK1 3’ untranslated region and is involved in upregulating PLK1 expression and that demethylation of this site activates the ataxia telangiectasia and Rad3-related protein pathway by replicating stress and increasing mitotic catastrophe, resulting in increased radiosensitivity.	2022	[90]
	METTL3	Writer	Oncogene	Downregulation	NUCB1	METTL3-mediated m(6)A modification on NUCB1 5’UTR via YTHDF2 as a mechanism for NUCB1 downregulation in PDAC. By controlling ATF6 activity, NUCB1 overexpression suppressed GEM-induced UPR and autophagy.	2021	[91]
	METTL3	Writer	Oncogene	Upregulation	miR-25-3p	Cigarette smoke condensate (CSC) caused the hypomethylation of the METTL3 promoter. Overexpressed METTL3 catalyzed the maturate of primary miR-25, which is mediated by NKAP. Mature miR-25, miR-25-3p, suppresses PHLPP2, resulting in the activation of oncogenic AKT-p70S6K signaling, which provokes malignant phenotypes of pancreatic cancer cells.	2019	[92]
	METTL3	Writer	Oncogene	Upregulation	LIFR-AS1	METTL3 induced m(6)A hyper-methylation on the 3’ UTR of LIFR-AS1 to enhance its mRNA stability and LIFR-AS1 could directly interact with miR-150-5p, thereby indirectly upregulating VEGFA expressions within cells and impact VEGFA/PI3K/Akt signaling.	2021	[93]
	METTL5	Writer	Oncogene	Upregulation	c-Myc	m(6)A modifications at the 5’ untranslated region (5’UTR) and coding DNA sequence region (near the 5’UTR) of c-Myc mRNA played a critical role in the specific translation regulation by METTL5. In addition, METTL5 and its cofactor tRNA methyltransferase activator subunit 11-2 synergistically promote pancreatic cancer progression.	2022	[94]
	METTL14	Writer	Oncogene	Upregulation	CDA	The transcriptional factor p65 targeted the promoter region of METTL14 and upregulated its expression, which then increased the expression of CDA, an enzyme-inactivate gemcitabine. Furthermore, depletion of METTL14 rescue the response of the resistance cell to gemcitabine.	2021	[95]
	METTL14	Writer	Oncogene	Downregulation	PERP	METTL14 direct targeting of the downstream PERP mRNA (p53 effector related to PMP-22) in an m(6)A-dependent manner. Methylation of the target adenosine lead to increased PERP mRNA turnover, thus decreasing PERP (mRNA and protein) levels in pancreatic cancer cells, promoting the growth and metastasis of pancreatic cancer.	2020	[96]
	METTL14	Writer	Oncogene	Upregulation	CLK1/SRSF5	CLK1 enhanced phosphorylation on SRSF5(250-Ser), which inhibited METTL14(exon10) skipping while promoted cyclin L2(exon6.3) skipping. In addition, aberrant METTL14(exon10) skipping enhanced the N6-methyladenosine modification level and metastasis, while aberrant cyclin L2(exon6.3) promoted proliferation of PDAC cells.	2021	[97]
	IGF2BP2	Reader	Oncogene	Upregulation	lncPACERR/miR-671-3p	LncRNA-PACERR activate KLF12/p-AKT/c-myc pathway by binding to miR-671-3p. LncRNA-PACERR which binds to IGF2BP2 acts in an m6A-dependent manner to enhance the stability of KLF12 and c-myc in cytoplasm. In addition, the promoter of LncRNA-PACERR was a target of KLF12 and LncRNA-PACERR recruited EP300 to increase the acetylation of histone by interacting with KLF12 in nucleus.	2022	[98]
	IGF2BP2	Reader	Oncogene	Downregulation	miR-141	IGF2BP2 is a direct target of miR-141. A negative correlation between IGF2BP2 mRNA expression and the expression of miR-141. Moreover, upregulating IGF2BP2 expression promotes pancreatic cancer cell growth by activating the PI3K/Akt signaling pathway in vitro and in vivo.	2019	[99]
	FTO	Eraser	Oncogene	Downregulation	YTHDF1/TFPI-2	FTO promotes the progression of PC through reducing m(6)A/YTHDF1 mediated TFPI-2 mRNA stability.	2022	[100]
	FTO	Eraser	Oncogene	Upregulation	MYC and bHLH	FTO interact with MYC proto-oncogene, bHLH transcription factor and enhance its stability by decreasing its m(6)A level, which promotes growth and metastasis and regulates PDAC cells.	2019	[101]
	FTO	Eraser	Oncogene	Upregulation	YTHDF2/PDGFC	FTO directly targets PDGFC and stabilizes its mRNA expression in an m(6)A-YTHDF2-dependent manner. PDGFC upregulation led to reactivation of the Akt signaling pathway, promoting PC cell growth.	2022	[102]
	FTO	Eraser	Anti-oncogene	Upregulation	YTHDF2/PJA2	FTO demethylated the m6A modification of praja ring finger ubiquitin ligase 2 (PJA2), thereby reducing its mRNA decay, suppressing Wnt signaling, and ultimately restraining the proliferation, invasion, and metastasis of pancreatic cancer cells.	2021	[103]
	YTHDF1	Reader	Anti-oncogene	Downregulation	PHF10	PHF10 was found and involved in the DNA damage response. PHF10 loss-of-function resulted in elevated recruitment of γH2AX, RAD51, and 53BP1 to DSB sites and decreased HR repair efficiency. Moreover, ZC3H13 knockdown downregulated the m(6)A methylation of PHF10 and decreased PHF10 translation in a YTHDF1-dependent manner.	2022	[104]
	YTHDF3	Reader	Oncogene	Downregulation	DICER1-AS1	YTHDF3 was a critical target for miR-5586-5p, forming negative feedback with DICER1-AS1. The negative feedback of YTHDF3 and glycolytic lncRNA DICER1-AS1 is involved in glycolysis and tumorigenesis of PC.	2022	[105]
	WTAP	Writer	Oncogene	Upregulation	WTAPP1	m6A modification stabilized WTAPP1 RNA via CNBP, resulting in increased levels of WTAPP1 RNA in PDAC cells. Excessive WTAPP1 RNA bound its protein-coding counterpart WTAP mRNA and recruited more EIF3 translation initiation complexes to promote WTAP translation. Increased WTAP protein enhanced the activation of Wnt signaling and provoked the malignant phenotypes of PDAC.	2021	[106]
	YTHDC1	Reader	Anti-oncogene	Upregulation	miR-30d/RUNX1	YTHDC1 facilitated the biogenesis of mature miR-30d via m(6)A-mediated regulation of mRNA stability. Then, miR-30d inhibited aerobic glycolysis through regulating SLC2A1 and HK1 expression by directly targeting the transcription factor RUNX1, which bound to the promoters of the SLC2A1 and HK1 genes.	2021	[107]
	ALKBH5	Eraser	Anti-oncogene	Upregulation	YTHDF2/PER1	ALKBH5 post-transcriptionally activated PER1 by m6A demethylation in an m6A-YTHDF2-dependent manner. PER1 upregulation led to the reactivation of ATM-CHK2-P53/CDC25C signalling, which inhibited cell growth. P53-induced activation of ALKBH5 transcription acted as a feedback loop regulating the m6A modifications in PC.	2020	[108]
	ALKBH5	Eraser	Anti-oncogene	Upregulation	KCNK15-AS1	ALKBH5 was downregulated in cancer cells, which can demethylate KCNK15-AS1 and regulate KCNK15-AS1-mediated cell motility to inhibit pancreatic cancer motility.	2018	[109]
	ALKBH5	Eraser	Anti-oncogene	Upregulation	KCNK15-AS1	ALKBH5 was verified to induce m(6)A demethylation of KCNK15-AS1 to mediate KCNK15-AS1 upregulation. KCNK15-AS1 combined with KCNK15 5’UTR to inhibit KCNK15 translation. Moreover, KCNK15-AS1 recruited MDM2 to promote REST ubiquitination, thus transcriptionally upregulating PTEN to inactivate AKT pathway.	2021	[110]
	ALKBH5	Eraser	Anti-oncogene	Downregulation	IRP2 and SNAI1	Owing to FBXL5-mediated degradation, ALKBH5 overexpression incurred a significant reduction in iron-regulatory protein IRP2 and the modulator of EMT SNAI1. ALKBH5 overexpression led to a significant reduction in intracellular iron levels as well as cell migratory and invasive abilities.	2021	[111]
	ALKBH5	Eraser	Anti-oncogene	Downregulation	WIF-1	Silencing ALKBH5 is correlated with WIF-1 transactivation and mediation of the Wnt pathway and increases PDAC cell proliferation, migration, and invasion.	2020	[112]
Pituitary Somatotroph Adenomas	METTL3	Writer	Oncogene	Upregulation	GNAS and GADD45γ	m6A methyltransferase METTL3 promotes tumor growth and hormone secretion by increasing expression of GNAS and GADD45γ in a m6A-dependent manner.	2022	[113]
